# Modelling longitudinal binary outcomes with outcome dependent observation times: an application to a malaria cohort study

**DOI:** 10.1186/s12936-022-04386-1

**Published:** 2022-12-10

**Authors:** Adelino Martins, Sereina A. Herzog, Levicatus Mugenyi, Christel Faes, Niel Hens, Steven Abrams

**Affiliations:** 1 Interuniversity Institute for Biostatistics and statistical Bioinformatics, Data Science Institute, UHasselt, Diepenbeek, Belgium; 2 Department of Mathematics and Informatics, Eduardo Mondlane University, Maputo, Mozambique; 3 Institute for Medical Informatics, Statistics and Documentation (IMI), Medical University of Graz, Graz, Austria; 4 Centre for Health Economics Research and Modelling Infectious Diseases, Vaccine and Infectious Disease Institute, University of Antwerp, Antwerp, Belgium; 5 Family Medicine and Population Health, University of Antwerp, Antwerp, Belgium; 6 Infectious Diseases Research Collaboration, Plot 2C Nakasero Hill road, Kampala, Uganda

**Keywords:** Interval censored data, Malaria infection, Markov Chain Monte Carlo (MCMC), Right-truncation, Time at risk

## Abstract

**Background:**

In spite of the global reduction of 21% in malaria incidence between 2010 and 2015, the disease still threatens many lives of children and pregnant mothers in African countries. A correct assessment and evaluation of the impact of malaria control strategies still remains quintessential in order to eliminate the disease and its burden. Malaria follow-up studies typically involve routine visits at pre-scheduled time points and/or clinical visits whenever individuals experience malaria-like symptoms. In the latter case, infection triggers outcome assessment, thereby leading to outcome-dependent sampling (ODS). Commonly used methods to analyze such longitudinal data ignore ODS and potentially lead to biased estimates of malaria-specific transmission parameters, hence, inducing an incorrect assessment and evaluation of malaria control strategies.

**Methods:**

In this paper, a new method is proposed to handle ODS by use of a joint model for the longitudinal binary outcome measured at routine visits and the clinical event times. The methodology is applied to malaria parasitaemia data from a cohort of $n = 988$ Ugandan children aged 0.5–10 years from 3 regions (Walukuba—300 children, Kihihi—355 children and Nagongera—333 children) with varying transmission intensities (entomological inoculation rate equal to 2.8, 32 and 310 infectious bites per unit year, respectively) collected between 2011–2014.

**Results:**

The results indicate that malaria parasite prevalence and force of infection (FOI) increase with age in the region of high malaria intensity with highest FOI in age group 5–10 years. For the region of medium intensity, the prevalence slightly increases with age and the FOI for the routine process is highest in age group 5–10 years, yet for the clinical infections, the FOI gradually decreases with increasing age. For the region with low intensity, both the prevalence and FOI peak at the age of 1 year after which the former remains constant with age yet the latter suddenly decreases with age for the clinically observed infections.

**Conclusion:**

Malaria parasite prevalence and FOI increase with age in the region of high malaria intensity. In all study sites, both the prevalence and FOI are highest among previously asymptomatic children and lowest among their symptomatic counterparts. Using a simulation study inspired by the malaria data at hand, the proposed methodology shows to have the smallest bias, especially when consecutive positive malaria parasitaemia presence results within a time period of 35 days were considered to be due to the same infection.

**Supplementary Information:**

The online version contains supplementary material available at 10.1186/s12936-022-04386-1.

## Background

Malaria infections are caused by *Plasmodium* parasites that are transmitted through bites of infected female mosquitoes and is potentially life-threatening. In spite of the fact that malaria is a preventable and mostly curable disease for which increased efforts worldwide dramatically reduced malaria incidence (i.e., a reduction of 21% between 2010 and 2015 as reported by WHO [1] and a further decrease thereof up to 2019 which was disrupted by the COVID-19 pandemic [2]), African countries still carry a disproportionately high share of the overall malaria burden. In order to reduce the malaria burden in African countries such as Uganda, a correct assessment and evaluation of the impact of control strategies is quintessential. Measures of malaria transmission intensity such as the entomological inoculation rate (EIR), the parasite prevalence and the malaria force of infection (FOI) have been used frequently to quantify the impact of various interventions [3, 4]. In general, malaria transmission has been reported to be highly inefficient, meaning that the ratio of EIR to FOI is relatively high. As is the case for other infections, individual- and household-specific heterogeneity in malaria acquisition is hardly ever accounted for in the estimation of the aforementioned epidemiological parameters, albeit that it is well-recognized that variability in environmental and host-related factors, among other sources, has an important effect thereon [5].

Often in clinical trials with follow-up to study (infectious) disease dynamics, study participants are asked to come to the clinic and get examined for malaria infection during scheduled (routine) visits. On top of that, unscheduled (clinical) visits can occur when participants develop symptoms for the disease under consideration, or when they experience symptoms similar to those typically observed for the infection at hand. If infection triggers outcome assessment in between pre-scheduled follow-up visits, the outcome and observation-time processes are said to be dependent, which in literature is often referred to as biased sampling or outcome-dependent sampling (ODS) [6]. Conventional longitudinal methods to analyze repeated measurements for subjects over time assume independence of both processes. Hence, such unscheduled visits, and the ODS they induce, could lead to biased estimation of the epidemiological quantities of interest when not appropriately accounted for in the statistical analysis.

Different models have been proposed to address ODS (also referred to as irregular observation times in longitudinal data) in both prospective experimental settings as well as retrospective observational studies. One commonly used method to deal with ODS is to conduct a weighted analysis in which weights are inversely proportional to the probability of being sampled (IPW; [7]). Alternatively, a weighted pseudo-likelihood method could be considered [8]. However, these methods rely on approximating the sampling scheme. Zhou et al. [9] proposed a semiparametric empirical likelihood(-based) method for an ODS scheme with a continuous outcome variable, in which the distribution of exposure variables is treated as nuisance parameter and is left unspecified. ODS designs for longitudinal binary responses also have been discussed in the literature in the context of case-control sampling [10] and time-varying covariates [11], either with (biased) sampling based on individual measurements, defining cases and controls, or based on a combination of the individual’s repeated measurements (i.e., through the inclusion of subjects with a response profile being sufficiently variable). Conditional and marginal models have been proposed in this context to adjust the analysis for the sampling mechanism to ensure valid statistical inference. On the other hand, Ryu et al. [12] considered longitudinal studies in which the measurement time points were unequally spaced and having a follow-up measurement at any time depend on the history of past visits and outcomes of that individual. These authors discussed limitations of previously proposed models and methods for longitudinal data, such as generalized linear mixed models and generalized estimating equations (GEE), which do not address the association between the outcome and observation time process. Furthermore, these authors proposed a joint model using latent random variables in which the observed follow-up times are described together with the longitudinal response data [12]. More recently, Tan [6] considered a joint model with a semiparametric regression model for the longitudinal outcomes and a recurrent event model for the observation times. Rizopoulos et al. [13] stated that an attractive paradigm for the joint modelling of longitudinal and time-to-event processes is the shared parameter framework [14] in which a set of random-effects is assumed to describe the interdependence of the two processes. For a review of methods to analyze longitudinal data subject to irregular observation times, the work by Pullenayegum and Lim [15] is a comprehensive source.

Although several authors developed methods to accommodate ODS in various settings, in this paper, a new method is proposed to cope with both the routine and clinical data on malaria infections from a cohort study in Uganda. To be more specific, a joint model for longitudinal binary outcomes (routine process) and time-to-event data (clinical process) is considered, as proposed earlier by Rizopoulos et al. [13], however, explicitly linking the processes through a similar functional form for the process-specific baseline forces of infection, and accommodating individual- and household-specific heterogeneity in the acquisition of malaria infections. Moreover, the proposed event-time model accounts for complexities in the data including truncation and interval-censoring. Furthermore, the statistical model is linked with a mathematical SIS compartmental model to mimic the natural history of malaria infection and to allow for the estimation of the force of infection based on a model for the parasite prevalence [5]. This paper focuses on the estimation of the malaria parasite prevalence in three regions of Uganda, accounting for observed and unobserved heterogeneity as was done previously, while dealing with ODS at the same time. A joint modelling approach is considered in which longitudinal binary outcome data collected during scheduled routine visits is linked to time-to-event data from the same subjects, if present, obtained during unscheduled clinical visits. More specifically, individual- and household-specific random effects will be specified in both processes to account for dependence.

The paper is organized as follows. A motivating example is introduced and briefly discussed in the following section. The general methodology to estimate malaria FOI from parasitaemia data is presented next. The impact of ignoring ODS is highlighted through a simulation study after which the proposed joint model is fitted to the available routine and clinical data on parasite presence in Ugandan children. Finally, the results are discussed in the Discussion section together with strengths and limitations of the proposed methodology.

## Motivating example

**Fig. 1:**
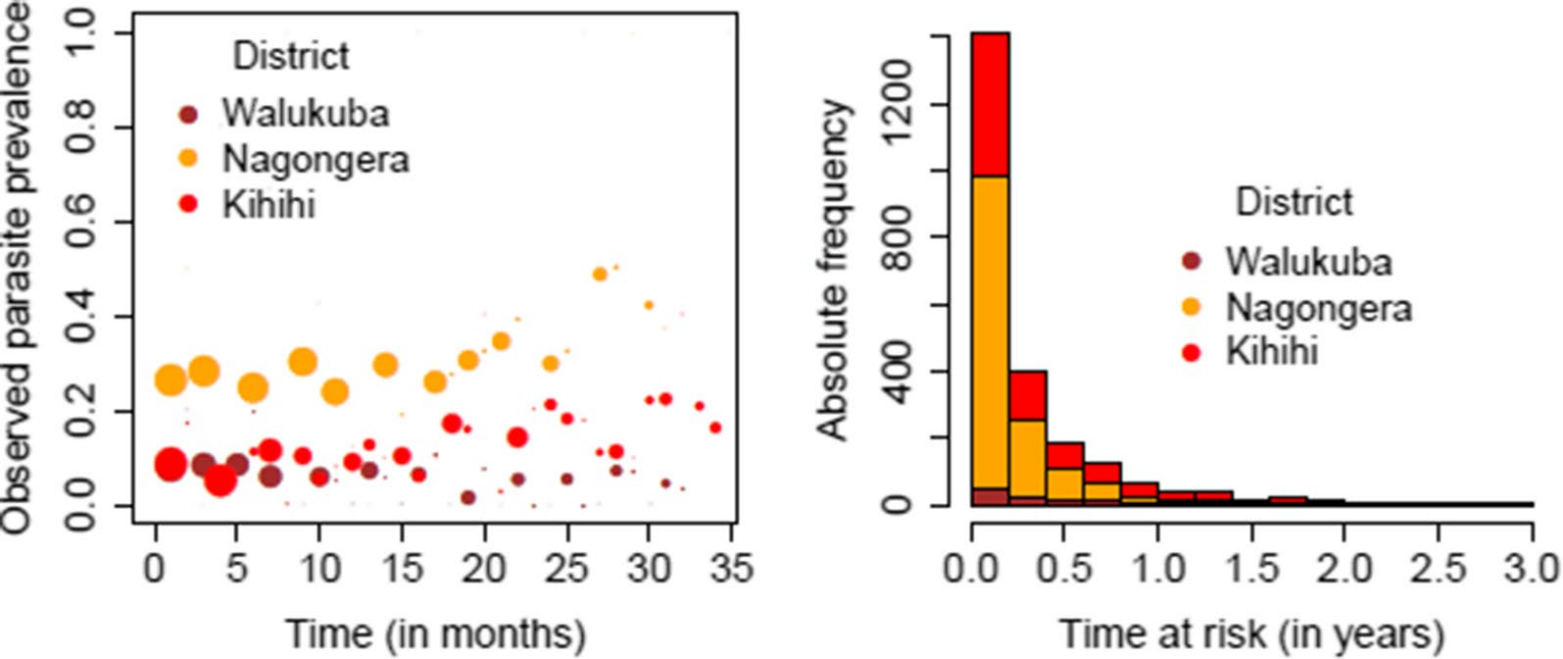
Observed parasite prevalence by time (in months) since the start of the study based on routine data collected in Walukuba (brown), Kihihi (red) and Nagongera (orange; left panel), and stacked histogram of the time at risk in years (times to symptomatic malaria infection) obtained from clinical visits in Walukuba (brown), Kihihi (red) and Nagongera (orange, right panel). In the left panel, the size of the points is proportional to number of observations

In this paper, longitudinal cohort data from children aged 0.5 to 10 years in three regions in Uganda (Nagongera sub-county in the district of Tororo; Kihihi sub-county in the district of Kanungu; and Walukuba sub-county in the district of Jinja) are considered. The data were collected as part of the Program for Resistance, Immunology, Surveillance and Modelling of malaria (PRISM) study [4]. More specifically, the PRISM data considered in this paper belongs to Phase I of the project conducted between August 2011 and September 2017 [4, 16]. The aforementioned study regions were characterized by distinct transmission intensities, with the highest intensity reported in Nagongera, followed by Kihihi and with Walukuba having the smallest intensity [4, 5], at least in Phase 1 of the study. The study participants were recruited from 300 randomly selected households (100 households from each region). In total, $n = 988$ children were followed over time with 300 children in Walukuba, 355 in Kihihi and 333 children in Nagongera. Individuals were routinely tested for the presence of *Plasmodium* parasites using microscopy every three months from August 2011 to August 2014 (3 years). Furthermore, tests were also conducted at unscheduled clinical visits whenever individuals experience malaria-like symptoms. In the left panel of Fig. 1, the observed parasite prevalence based on the collected data in Nagongera (orange), Kihihi (red) and Walukuba (brown) is graphically depicted. A stacked histogram showing the frequency of times at risk for symptomatic malaria infections can be found in the right panel of Fig. 1. Nagongera has the highest observed parasite prevalence and the highest number of symptomatic malaria infections followed by Kihihi and Walukuba. After a child cleared the malaria parasites, the time at risk for symptomatic malaria infections is the time that it takes to experience the next infection. In the PRISM study, the time to the second, third or the *n*-th infection is only known to lie between the point the child is tested positive and the point he/she first tested negative after recovering from the previous infection. More detailed information regarding the study design can be found in Kamya et al. [4].

Throughout this paper, the outcome process refers to the occurrence of the longitudinal binary outcome (parasite presence), and the observation-time process relates to the timing of scheduled, i.e., routine, and unscheduled, i.e., clinical, visits over the entire follow-up period of the study.

## Materials and methods

### Malaria dynamics—a simplified transmission model

For the purpose of this paper, a simplified version of a realistic transmission model to describe malaria infection dynamics is considered. More specifically, following Mugenyi et al. [5], a so-called Susceptible (S) - Infected (I) - Susceptible (S), or short SIS, compartmental model dividing the population into two mutually exclusive compartments will be used to describe malaria dynamics within the human host. For a detailed discussion on the motivation for the choice of the SIS model, the reader is referred to Mugenyi et al. [5]. Note that the methodology outlined here is more generally applicable in the case of other disease dynamics. Herein, as defined in [5] let *S*(*a*) denote the proportion of susceptible individuals in the population and *I*(*a*) the proportion of infected individuals of age *a*, i.e., the (point) parasite prevalence, then the following set of ordinary differential equations (ODEs) describes transitions in the compartmental SIS model [5]:

1 $$\begin{aligned} \begin{aligned} S^{'}(a)&=-\lambda (a)S(a) + \gamma I(a), \\ I^{'}(a)&=\lambda (a)S(a) - \gamma I(a), \end{aligned} \end{aligned}$$

where $\gamma$ represents a time- and age-invariant clearance rate at which individuals regain susceptibility after clearing malaria parasites from their blood. Hence, the FOI $\lambda (a)$ which represents the instantaneous rate at which individuals move from the susceptible compartment S to the infected compartment I at age *a* (i.e., the age-specific rate at which individuals are infected with malaria parasites through effective mosquito bites) can easily be derived in terms of the point prevalence *I*(*a*) [5]:

2 $$\begin{aligned} \lambda (a) = \frac{I'(a) + \gamma I(a)}{1 - I(a)}, \end{aligned}$$

using $I(a) + S(a)=1$. Hence, constructing a model for the point prevalence *I*(*a*) yields the FOI $\lambda (a)$ given the clearance rate $\gamma$.

### Model specification

First of all, the terminology and notation used to specify the proposed model to encompass ODS when modelling the data is introduced. Consider a sample of *n* individuals, denoted by individual $i = 1, \ldots , n$, in which individual *i* has a total of $n_i$ visits (i.e., routine or clinical visits as a result of recurrent malaria infections) which are indexed by $j = 1, \ldots , n_i$. Let $n_{i(r)}$ and $n_{i(c)}$ represent the number of routine and unscheduled clinical visits for individual *i*, respectively, implying that $n_i~=~n_{i(r)}~+~n_{i(c)}$. The models introduced in the following subsections consider: (1) $n_{i(r)}$ routine visits; (2) $n_{i(c)}$ unscheduled clinical visits; and (3) both routine and unscheduled clinical visits.

#### Parasite prevalence and routine visits

Consider the binary random variable $Y_{ij}$ representing an indicator for the presence of malaria parasites for individual *i* at (routine) visit *j*. Consequently, for scheduled routine visits, $\left( Y_{ij} | a_{ij}, \varvec{x}_i, \varvec{b}_i \right) \sim \text{ B }(I(a_{ij}|\varvec{x}_i, \varvec{b}_i))$ with $\text{ B }$ denoting the Bernoulli distribution, and where $a_{ij}$ represents the age of individual *i* at visit *j*, $\varvec{x}_i$ represents a $(p \times 1)$-vector of covariate information for individual $i = 1, \ldots , n$, and $\varvec{b}_i$ a $(q \times 1)$-vector of individual-specific random effects. In order to model the parasite prevalence, a generalized linear mixed model with $\textrm{cloglog}$-link is formulated as follows:

3 $$\begin{aligned} \begin{aligned} \textrm{cloglog}\left[\it I(a_{ij} | \varvec{x}_i, \varvec{b}_i)\right] = {\it h}(a_{ij}; \varvec{\theta }) + \varvec{\beta }^{T}\varvec{x}_i+\varvec{b}^{T}_i\varvec{z}_i, \end{aligned} \end{aligned}$$

where $\varvec{\beta }$ is a column vector of unknown regression parameters and $\varvec{z}_i$ is an individual-specific $(q \times 1)$ design vector for $\varvec{b}_i$ which is a column vector of individual-specific normally distributed random effects, i.e., $\varvec{b}_i \sim N(\varvec{\mu }, \varvec{D})$ thereby addressing the association among repeated measurements over time within the same individual. Here, the variance-covariance matrix $\varvec{D}$ is assumed to be a diagonal matrix with variances on the main diagonal. Moreover, $h(a_{ij}; \varvec{\theta })$ is a known function describing the age-effect with parameter vector $\varvec{\theta }$. Note that the calendar time effect can be introduced in the linear predictor by means of the shifted birth year of the *i*th individual, implying the prevalence, and equivalently the FOI, to depend on both age and calendar time [5]. In Table 1, some common parametric distributions are presented together with their implied functional forms for $h(a_{ij}; \varvec{\theta })$ based on model (3) and the corresponding baseline infection risk $\lambda _0(a_{ij}) = h'(a_{ij}; \varvec{\theta })\exp \left[ h(a_{ij}; \varvec{\theta })\right]$ (derived under the assumption of no parasite clearance).

In the absence of unscheduled clinical visits $n_i = n_{i(r)}$, or under the assumption of independence between the observation time process and the outcome process, the model parameters can either be estimated using maximum likelihood techniques or within the Bayesian framework using Markov Chain Monte Carlo (MCMC) methods, thereby maximizing a marginal likelihood function with the following individual conditional likelihood contributions:

4 $$\begin{aligned} \begin{aligned} L_{1i}({\varvec{\Psi }}| \varvec{y}_{i}, a_{ij}, \varvec{x}_i, \varvec{b}_i)&= \prod _{j = 1}^{n_{i(r)}}I(a_{ij}|\varvec{x}_i, \varvec{b}_i)^{y_{ij}} \\&\quad \times \left[ 1 - I(a_{ij}|\varvec{x}_i, \varvec{b}_i)\right] ^{(1 - y_{ij})}, \end{aligned} \end{aligned}$$

with ${\varvec{\Psi }}=(\varvec{\beta }, \varvec{\theta })^T$, and where $y_{ij}$ is the observed binary outcome for individual *i* at routine visit $j=1, \ldots , n_{i(r)}$, and $I(a_{ij} | \varvec{x}_i, \varvec{b}_i)$ is the conditional parasite prevalence. In the following subsection, the focus is specifically on clinical visits and how to address ODS.

**Table 1:** Distributional assumptions regarding the underlying age-specific malaria FOI

Distribution	$\varvec{\theta }$	$h(a_{ij}; \varvec{\theta })$	$\lambda _0(a_{ij})$
Exponential	$\theta _1 > 0$	$\log (\theta _1 a_{ij})$	$\theta _1$
Weibull	$\theta _1, \theta _2 > 0$	$\log (\theta _1 a_{ij}^{\theta _2})$	$\theta _1 \theta _2 a_{ij}^{\theta _2-1}$
Gompertz	$\theta _1 > 0, -\infty< \theta _2 < +\infty$	$\log \left[ \frac{\theta _1}{\theta _2} \left( e^{\theta _2 a_{ij}}-1\right) \right]$	$\theta _1 e^{\theta _2 a_{ij}}$
Log-logistic	$\theta _1, \theta _2 > 0$	$\log \left\{ \log \left[ 1+\left( \theta _1 a_{ij}\right) ^{\theta _2}\right] \right\}$	$\frac{\theta _1 \theta _2 (\theta _1 a_{ij})^{\theta _2 - 1}}{1 + (\theta _1 a_{ij})^{\theta _2}}$
Fractional polynomial	$\theta _2 < 0$	$\theta _2 a_{ij}^{-1}$	$-\theta _2 a_{ij}^{-2} e^{\theta _2 a_{ij}^{-1}}$

#### Outcome-dependent sampling and clinical visits

As mentioned before, clinical visits due to symptomatic malaria infections, or malaria-like events giving rise to symptoms similar to those observed for malaria, can not be treated in the same way as described previously. Let $t^*_{ij}$ represent the true time at risk for an individual *i* for which the *j*th visit is clinical, and $c_{ij}$ an indicator variable such that $c_{ij}~=~1$ if the individual comes to the clinic during an unscheduled clinical visit and $c_{ij}~=~0$ for a scheduled (routine) visit. For illustration purposes, $t^*_{ij}$ is assumed to be known, albeit that this is not the case in practice, and statistical ways to deal with this are outlined below.

During a clinical visit, either a symptomatic malaria infection or a malaria-like event will cause the individual to have symptoms and to visit the health center. In the analysis, only symptomatic malaria infections are considered. Let $f^*_T$ and $S^*_T$ denote the probability density and survival function for $T^*_{ij}$, respectively, then these functions can be expressed in terms of the conditional age- and time-dependent infection risk $\lambda ^s(a|\varvec{b}_i)$, suppressing dependence on $\varvec{x}_i$, as follows:

$$\begin{aligned} \begin{aligned} f^{*}_T(t^*_{ij}|a_{ij}, \varvec{b}_i)&= \lambda ^s(a_{ij} + t^*_{ij}|\varvec{b}_i)e^{-\int _{a_{ij}}^{a_{ij} + t^*_{ij}}{\lambda ^s(u|\varvec{b}_i)du}},\\ S^*_T(t^*_{ij}|a_{ij}, \varvec{b}_i)&= e^{-\int _{a_{ij}}^{a_{ij} + t^*_{ij}}{\lambda ^s(u|\varvec{b}_i)du}}, \end{aligned} \end{aligned}$$

where $$a_{ij}$$ is the individual’s age at the time that he/she becomes susceptible to malaria infection related to the *j*th clinical visit and $$\lambda ^s(u|\varvec{b}_i) \equiv \lambda ^s(u|\varvec{x}_i, \varvec{b}_i)$$ $$= e^{\varvec{b}'_i \varvec{z}_i + \varvec{\zeta }' \varvec{x}_i}\lambda _0^{s}(u)$$ is the conditional age- and time-varying symptomatic malaria FOI under the proportional hazards assumption (with $$\varvec{\zeta }$$ a vector of model parameters). Note that $$S^*(t^*_{ij} | a_{ij}, \varvec{b}_i)$$ represents the probability of not experiencing a symptomatic infection in the interval $$[a_{ij}, a_{ij} + t^*_{ij})$$ and the instantaneous risk of being infected when being at risk for $$t^*_{ij}$$ units $$\lambda ^*(t^*_{ij} | a_{ij}, \varvec{b}_i)$$, conditional on becoming susceptible to (symptomatic) infection at age $$a_{ij}$$, equals the age-specific FOI $$\lambda ^s(a_{ij} + t^*_{ij} |\varvec{b}_i)$$ at current age $$a_{ij} + t^*_{ij}$$.

Although some symptomatic infection times $T^*_{ij}$ can be observed, those infection times exceeding the time at which the study ends $t_E$ are unobserved. Hence, the distribution of observed infection times, say $T_{ij}$, is truncated with probability density function

$$\begin{aligned} f_T(t_{ij} | a_{ij}, \varvec{b}_i) = \frac{f^*_T(t_{ij} | a_{ij}, \varvec{b}_i)}{1 - S^*_T(t_{Tij} | a_{ij}, \varvec{b}_i)}, \end{aligned}$$

where the individual-specific truncation time is equal to $t_{Tij} = t_E - a_{ij}$.

Different distributional assumptions can be made regarding the time at risk distribution, such as, e.g., exponential, Weibull, Gompertz, among others, which also relates to the selected functional form for $h(a_{ij}; \varvec{\theta })$ in the outcome process model (see Table 1). In order to align the models for both processes, the baseline infection risk $\lambda _0^{s}(u)$ for the observation time process can be of the same type as $\lambda _0(u)$, albeit that distributional parameters, say $\varvec{\vartheta }$, are allowed to be different. Note that more flexible parametric shapes for $h(a_{ij}; \varvec{\theta })$, such as, e.g., using fractional polynomials, could result in non-standard non-negative distributions for the malaria infection times, albeit that unconstrained optimization could lead to negative FOI estimates. In the statistical analysis, parametric fractional polynomials are included as an alternative to the standard event time distributions.

For outcomes $(\varvec{t}_{i(c)}, \varvec{y}_{i(c)})$ that are derived from the clinical visits $j = 1, \ldots$, $n_{i(c)}$, the conditional likelihood function has contributions:

5 $$\begin{aligned} \begin{aligned} L_{2i}({\varvec{\Theta }}|\varvec{t}_{i(c)}, \varvec{y}_{i(c)}, \varvec{a}_{i(c)}, \varvec{x}_i, \varvec{b}_i) = \prod _{j = 1}^{n_i(c)}{f_T}, \end{aligned} \end{aligned}$$

with ${\varvec{\Theta }}=(\varvec{\zeta }, \varvec{\vartheta })^T$, $\varvec{t}_{i(c)}$ and $\varvec{a}_{i(c)}$ are the vectors of time at risk and age of the individual at risk for the *j*th clinical event, respectively, and

$$\begin{aligned} f_T~:=~f_T(t_{ij(c)}|a_{ij(c)}, y_{ij(c)}, \varvec{x}_i, \varvec{b}_i). \end{aligned}$$

#### Combined model

Finally, the likelihood for the joint model including both information on routine and clinical visits is obtained by combining conditional likelihood contributions as described before:

$$\begin{aligned}&L_{3i}({\varvec{\Psi }}, {\varvec{\Theta }}|\varvec{t}_{i}, \varvec{y}_i, \varvec{a}_{i}, \varvec{x}_i, \varvec{c}_{i}, \varvec{b}_i) \\&\quad = \prod _{j = 1}^{n_i}{f(y_{ij}|a_{ij}, \varvec{x}_i, \varvec{b}_i)^{1 - c_{ij}}} \times f_T(t_{ij}|a_{ij}, y_{ij}, \varvec{x}_i, \varvec{b}_i)^{c_{ij}}, \end{aligned}$$

at least under the assumption that each malaria event contributes solely to one of the two components (i.e., routine or clinical process) in the likelihood. As mentioned previously, the time at risk for a specific clinical event (i.e., a symptomatic malaria infection) is not precisely known. More specifically, malaria infection times are interval-censored which needs to be taken into account in the statistical analysis through the modification of the likelihood function. For more details on how the interval-censoring has been treated in the analysis, the reader is referred to Additional file 1: Appendix B.1.

### Bayesian inference method

Following the development in the previous subsections, parameter estimation for the proposed combined model was performed in a Bayesian framework using MCMC sampling. The joint posterior distribution for the proposed models is then given by

6 $$\begin{aligned} P_{q}({\varvec{\Psi }},{\varvec{\Theta }},\varvec{b}_i|\varvec{O}_i)&\propto \left[ \prod _{i=1}^{n} L_{qi}({\varvec{\Psi }}, {\varvec{\Theta }} | \varvec{O}_i, \varvec{b}_i) \right] \\&\quad \times \left[ \prod _{i=1}^{n} P(\varvec{b}_i|\varvec{\mu },\varvec{D})\right] P({\varvec{\Psi }})P({\varvec{\Theta }}), \end{aligned}$$

with $\varvec{O}_i=\{\varvec{t}_i,\varvec{y}_i,\varvec{a}_i,\varvec{x}_i,\varvec{c}_i\}$ and $q=1,2,3$ defining the model. The prior distributions for the model parameters in (6) are chosen to make the distribution proper but diffuse with large variances. In addition, the priors for all parameters are assumed independent such that the joint prior density equals the product of the marginal prior distributions.

Vague normal priors with mean zero and variance equal to 1000 are considered for all model parameters with support $]-\infty ,+\infty [$. A gamma prior distribution with unit mean and variance 100 was assumed for all unknown parameters with support $]0,+\infty [$. For the purpose of model comparison or selection, the widely applicable information criterion (WAIC) was used to estimate out-of-sample predictive accuracy [17,23, 24]. MCMC samples of the joint posterior distribution of the model parameters are obtained using Gibbs sampling via the JAGS function and the R2jags package [18] implemented in the statistical software package R version 4.1.3 (R Development Core Team 2022). The R program to fit the proposed model can be found in the Supporting Materials.

## Simulation study

To investigate the impact of ignoring ODS, a simulation study that is inspired by the PRISM data under consideration is conducted. More specifically, as a Bayesian approach is time-consuming, only $M = 100$ datasets, including $n_m \equiv n = 1000$ individuals per simulated dataset (denoted by $m = 1, \ldots , M$) are generated. In particular, datasets were generated in order to accommodate the four different scenarios corresponding to the conditional log-likelihood functions as previously described. An overview of these scenarios is provided in Table 2. Scenario 1 and 3 make use of routine and clinical data, respectively, whereas Scenario 2 utilizes both routine and clinical data, but ignores ODS. In Scenario 4 the log-likelihood function uses both routine and clinical data and takes ODS into account. Furthermore, a simulation setting in which exponential infection times occur during a follow-up period of 1800 days ($\approx$ 5 years) and with an average duration until acquiring a new infection of about 365 days (1 year: $\lambda _0 = \lambda ^*_0 = \exp (-5.9) = 0.0027$) is considered. Parasite clearance times are exponentially distributed with a mean duration of infectiousness equal to 50 days ($\gamma = 0.02$). Based on the generated infection histories for the individuals, routine and clinical visits are obtained. More specifically, routine visits are scheduled every 90 days and parasite presence is recorded based on the current status at the time of data collection. Varying probabilities for having a symptomatic malaria episode are considered in the simulation whereby symptomatic observations at unscheduled time points were considered as clinical visits (i.e., $P = 20\%, 40\%, 60\%, 80\%, 100\%$). Hence, asymptomatic malaria cases were only included when detected during the routine process. No malaria-like events were generated such that all clinical visits are due to symptomatic malaria infections. Individual-specific random intercepts $b_i \sim N(\mu , \sigma _b^2)$, $i = 1, \ldots , n$, with $\mu = -\sigma _b^2/2$ implying a unit mean for the log-normal random terms $e^{b_i}$, are introduced to induce correlation between repeated measurements for the same subject ($\sigma _b^2 = 0.25$).

**Table 2:** Overview of the different scenarios, corresponding log-likelihood functions and parasitaemia data that is included in the analyses

Scenario	Log-likelihood function	Parasitaemia data
1	$ll_{1}(\varvec{\beta }, \varvec{\theta } | \varvec{y}, \varvec{X}) = \sum _{i = 1}^{n}{\log \left[ L_{1j}(\varvec{\beta }, \varvec{\theta } | \varvec{y}_{i}, \varvec{x}_i)\right] }$	Routine
2	$ll_{1}(\varvec{\beta }, \varvec{\theta } | \varvec{y}, \varvec{X}) = \sum _{i = 1}^{n}{\log \left[ L_{1j}(\varvec{\beta }, \varvec{\theta } | \varvec{y}_{i}, \varvec{x}_i)\right] }$	Routine & clinical$^{*}$
3	$ll_{2}(\varvec{\zeta }, \varvec{\vartheta } | \varvec{t}, \varvec{y}, \varvec{a}, \varvec{X}) = \sum _{i = 1}^{n}{\log \left[ L_{2i}(\varvec{\zeta }, \varvec{\vartheta } | \varvec{t}_{i}, \varvec{y}_i, \varvec{a}_{i}, \varvec{x}_i)\right] }$	Clinical
4	$ll_{3}(\varvec{\beta }, \varvec{\theta }, \varvec{\zeta }, \varvec{\vartheta } | \varvec{t}, \varvec{y}, \varvec{a}, \varvec{X}) = \sum _{i = 1}^{n}{\log \left[ L_{3i}(\varvec{\beta }, \varvec{\theta }, \varvec{\zeta }, \varvec{\vartheta } | \varvec{t}_{i}, \varvec{y}_i, \varvec{a}_{i}, \varvec{x}_i)\right] }$	Routine & clinical

$^{*}$ Scenario 2 does not take ODS into account

An important point is that if a single infection is contributing to both the routine and clinical process (i.e., consecutive observations for positive result at clinical $C^{+}$, and routine $R^{+}$ visits, or vice versa), hence leading to two dependent observations, the second one in Scenario 4 is dropped. However, without additional information, it is difficult to determine whether individuals already recovered and got re-infected in between such visits, thereby potentially underestimating the FOI. A sensitivity analysis is performed given the simulation scenario at hand in order to deduce the time period in which consecutive positive routine and clinical observations can be considered to be the result of a single malaria infection. From this exercise, a period of 35 days is assumed to be optimal (see Additional file 1: Appendix A, Figure A.1 for more details thereon). This observation is supported by the literature where 100% recovery rate was reported on day 28 following anti-malaria treatment [19, 20].

### Simulation results

Hereunder, the results from fitting the four scenarios to the simulated data are presented based on the three different likelihoods, as previously described. The model simulations are carried out based on the four different scenarios, as shown in Table 2. The simulation results (based on a single MCMC chain of 10,000 iterations with thinning of four while discarding the initial 1500 iterations as burn-in; convergence was checked) with varying percentages of symptomatic malaria infections are summarized in Table 3 in terms of summary statistics of the posterior distributions (for each simulation run) for the model parameters. Scenario 2 including both routine and clinical data without accounting for ODS performs worse as compared to Scenario 1 in which only routine data is used. Hence, ignoring ODS leads to biased estimates of both the baseline hazard as well as population-averaged hazard functions. Note that Scenario 1 is not influenced by the percentage of symptomatic infections as clinical infections are not accounted for therein. The proposed model for the analysis of both clinical and routine parasitaemia data (Scenario 4) outperforms Scenarios 1, 2 and 3 in terms of bias and precision (and consequently root mean square error (RMSE) and mean length of credibly intervals (MLCI)) for the baseline hazard function, population-averaged hazard $\lambda _p$, and variance of the random intercepts $\sigma ^2_{b}$, and leads in all cases to a reduction in bias. In Scenario 4, clinical information is added to the readily available routine data (i.e., larger sample size), resulting in a lower RMSE, bias and empirical variance for the model parameters as compared to Scenario 1.

**Table 3:** Simulation results for the different models showing the simulation-based average of the posterior mean (average of the estimated standard errors)(simulation-based standard errors) for the marginal or population-averaged FOI $\lambda$ and variance of the random intercepts $\sigma ^2_{b}$, its corresponding bias, root mean square error (RMSE) and mean length of credibly intervals (MLCI). P represents the percentage of symptomatic infections. True values: (1) $\lambda =0.0027$ and (2) $\sigma ^2_{b}=0.25$. Scenario 4 uses all data except for positive routine observations following a positive clinical visit, or positive clinical observations following a positive routine visit within a 35 day period. *N* represents the total number of observations over all individuals averaged over the *M* datasets

	Scenario 1	Scenario 2$^{*}$	Scenario 3	Scenario 4
	Routine data	Routine & clinical data	Clinical data	Routine & clinical data
P = 20%	N = 20,000	N = 21,687	N = 1687	N = 21,487
${\bar{\hat{\lambda }}}$	0.0027 (8.9e−5) (8.3e−5)	0.0036 (1.1e−4) (1.0e−4)	0.0032 (2.1e−4) (2.7e−4)	0.0027 (7.6e−5) (7.9e−5)
$\text{ Bias }(\lambda )$	5.80e−5	9.20e−4	5.60e−4	4.40e−5
$\text{ RMSE }(\lambda )$	1.00e−4	9.30e−4	6.20e−4	9.04e−5
MLCI	3.50e−4	4.30e−4	7.80e−4	3.00e−4
$\bar{\hat{\sigma ^2_{b}}}$	0.4086(0.0437)(0.0451)	0.4311 (0.0408) (0.0387)	0.2948 (0.0894) (0.0843)	0.3012 (0.0386) (0.0367)
$\text{ Bias }(\sigma ^2_{b})$	0.1586	0.1811	0.0448	0.0513
$\text{ RMSE }(\sigma ^2_{b})$	0.1650	0.1850	0.0955	0.0631
MLCI	0.1703	0.15947	0.4235	0.1500

P = 40%	N = 20,000	N = 22,534	N = 2534	N = 22,334
$\bar{\hat{\lambda }}$	0.0027 (8.9e−5) (8.3e−5)	0.0046 (1.3e−4) (1.2e−4)	0.0031 (1.4e−04) (1.6e−4)	0.0027 (7.2e−5) (7.6e−5)
$\text{ Bias }(\lambda )$	5.80e−5	1.90e−3	4.50e−4	4.00e−5
$\text{ RMSE }(\lambda )$	1.00e−4	2.00e−3	4.80e−4	8.85e−5
MLCI	3.50e−4	5.20e−4	5.30e−4	2.80e−4
$\bar{\hat{\sigma ^2_{b}}}$	0.4087 (0.0447) (0.0450)	0.4179 (0.0369) (0.0380)	0.2892 (0.0594) (0.0582)	0.2883 (0.0330) (0.0309)
$\text{ Bias }(\sigma ^2_{b})$	0.1587	0.1679	0.0392	0.0381
$\text{ RMSE }(\sigma ^2_{b})$	0.1650	0.1720	0.0702	0.0491
MLCI	0.1738	0.1439	0.2235	0.1276

P = 60%	N = 20,000	N = 23,376	N = 3376	N = 23,176
$\bar{\hat{\lambda }}$	0.0027 (8.9e−5) (8.4e−5)	0.0055 (1.6e−4) (1.5e−4)	0.0030 (1.0e−4) (1.1e−4)	0.0027 (6.9e−5) (7.0e−5)
$\text{ Bias }(\lambda )$	5.70e−5	2.80e−3	3.20e−4	2.15e−5
$\text{ RMSE }(\lambda )$	1.00e−4	2.80e−3	3.40e−4	7.32e−5
MLCI	3.50e−4	6.10e−4	4.10e−4	2.70e−4
$\bar{\hat{\sigma ^2_{b}}}$	0.4092 (0.0444) (0.0431)	0.4000 (0.0336) (0.0325)	0.2704 (0.0451) (0.0503)	0.2752 (0.0282) (0.0266)
$\text{ Bias }(\sigma ^2_{b})$	0.1592	0.1500	0.0204	0.0252
$\text{ RMSE }(\sigma ^2_{b})$	0.1650	0.153	0.0543	0.0366
MLCI	0.1734	0.1302	0.1716	0.1099

P = 80%	N = 20,000	N = 24,223	N = 4223	N = 24,077
$\bar{\hat{\lambda }}$	0.0027 (8.9e−5) (8.2e−5)	0.0065 (1.8e−4) (1.7e−4)	0.0029 (9.0e−5) (1.0e−4)	0.0027 (5.0e−5) (5.7e−5)
$\text{ Bias }(\lambda )$	5.80e−5	3.80e−3	2.40e−4	1.80e−5
$\text{ RMSE }(\lambda )$	1.00e−4	3.80e−3	2.60e−4	5.90e−5
MLCI	3.50e−4	6.80e−4	3.50e−4	1.90e−4
$\bar{\hat{\sigma ^2_{b}}}$	0.4094 (0.0436) (0.0425)	0.3829 (0.0307) (0.0312)	0.2624 (0.0375) (0.0395)	0.2469 (0.0233) (0.0235)
$\text{ Bias }(\sigma ^2_{b})$	0.1594	0.1329	0.0124	0.0031
$\text{ RMSE }(\sigma ^2_{b})$	0.1650	0.1365	0.0414	0.0237
MLCI	0.1698	0.1189	0.1448	0.0905

P = 100%	N = 20,000	N = 25,077	N = 5077	N = 24,977
$\bar{\hat{\lambda }}$	0.0027 (8.9e−5) (8.5e−5)	0.0075 (1.9e−4) (1.8e−4)	0.0028 (7.9e−5) (8.0e−5)	0.0027 (5.0e−5) (5.7e−5)
$\text{ Bias }(\lambda )$	6.00e−5	4.80e−3	1.40e−4	1.80e−5
$\text{ RMSE }(\lambda )$	1.00e−4	4.80e−3	1.60e−4	5.90e−5
MLCI	3.50e−4	7.50e−4	3.10e−4	1.80e−4
$\bar{\hat{\sigma ^2_{b}}}$	0.3984 (0.0434) (0.0449)	0.3640 (0.0286) (0.0299)	0.2499 (0.0323) (0.0332)	0.2489 (0.0210) (0.0203)
$\text{ Bias }(\sigma ^2_{b})$	0.1484	0.1140	6.10e−5	0.0011
$\text{ RMSE }(\sigma ^2_{b})$	0.1650	0.1178	0.0332	0.0203
MLCI	0.1695	0.1114	0.1204	0.0705

$^{*}$ Scenario 2 does not take ODS into account

## Data application

In this section, the proposed combined model (cfr. Scenario 4) is applied to the observed Ugandan malaria parasitaemia data as previously presented. In particular, different models are fitted in order to select important covariates and the most appropriate functional form for $h(a_{ij}; \varvec{\theta })$, and model comparison was done based on WAIC [17] as pointed out earlier. The covariates considered in the model building process included study site, age, shifted birth year (i.e., shifted birth year = birth year − birth year of the oldest child), previous use of artemether-lumefantrine (AL) treatment, and the infectious status at the previous visit. The covariate ‘shifted birth year’ is generated to represent the calendar time (see also [5] for details concerning this modelling strategy). Since malaria transmission intensity differs between the three sites (see, e.g., [4, 5]), site-stratified analyses are performed. Let *SID* represent the study site ID (1 = Walukuba, 2 = Kihihi, 3 = Nagongera), $a_{ij}$ the child’s age in years, $l_{ij}$ the shifted birth year, $PIS_{ij}$ the previous infection status and use of AL (1 = Negative & no AL, 2 = Negative + AL, 3 = Symptomatic, 4 = Asymptomatic) for individual *i* at visit *j*. Different parametric distributional assumptions regarding the infection times are explored (see Table 1) thereby allowing for different distributional parameters $\varvec{\theta }$ and $\varvec{\vartheta }$ for the outcome and infection time process, respectively.

One MCMC chain of total length 250,000 iterations is initiated with thinning factor = 15 and the first 40,000 iterations are removed as burn-in.

**Table 4:** Results obtained by fitting the proposed combined model, in which routine and clinical data are considered while taking into account ODS: Posterior estimates of model parameters from a converged MCMC chain for outcome process and observation time process models: posterior median and 95% highest posterior density (HPD) credible intervals

Effect	Parameter	Walukuba (N = 2483)	Kihihi (N = 4099)	Nagongera (N = 4123)
		Estimate (95% HPD)	Estimate (95% HPD)	Estimate (95% HPD)
Outcome process model				
Infection status at previous visit$^{*}$				
Negative+AL use	$\beta _1$	0.201 (− 0.279, 0.680)	− 0.303 (− 0.586, − 0.002)	− 0.419 (− 0.635, − 0.212)
Symptomatic	$\beta _2$	− 0.577 (− 1.489, 0.265)	− 0.942 (− 1.275, − 0.620)	− 1.042 (− 1.283, − 0.808)
Asymptomatic	$\beta _3$	1.513 (1.023, 1.997)	0.882 (0.527, 1.222)	0.416 (0.181, 0.643)
Shifted year of birth	$\beta _4$	− 0.059 (− 0.143, 0.025)	0.501 (0.372, 0.637)	0.277 (0.186, 0.367)
Age (Gompertz model)	$\theta _1$	0.061 (0.014, 0.123)	3.0e−4 (1.9e−5, 6.8e−4)	0.012 (0.002, 0.025)
	$\theta _2$	− 0.568 (− 0.966, − 0.211)	0.561 (0.410, 0.709)	0.224 (0.093, 0.355)
Observational time process model				
Shifted year of birth	$\zeta$	0.173 (0.084, 0.258)	0.125 (0.073, 0.179)	0.501 (0.408, 0.598)
Age (Gompertz model)	$\vartheta _1$	0.573 (0.188, 0.952)	0.753 (0.397, 1.126)	0.065 (0.011, 0.143)
	$\vartheta _2$	0.064 (− 0.022, 0.153)	0.202 (0.149, 0.257)	0.542 (0.440, 0.640)
Variance components				
Random intercept for households	$\sigma ^2_{b0}$	1.258 (0.653, 1.943)	1.198 (0.795, 1.620)	0.615 (0.401, 0.859)
Random intercept for subjects	$\sigma ^2_{b1}$	0.095 (0.002, 0.317)	0.233 (0.003, 0.656)	0.110 (0.002, 0.303)

$^{*}$ Reference category = Negative and no AL treatment in the past

Convergence of the chain is assessed using graphical diagnostic tools such as trace plots, histograms of posterior samples (representing the posterior density), and auto-correlation plots (see Additional file 1: Figures C.1-C.6 in Appendix C). Since the posterior distributions of some model parameters are skewed, the posterior medians are used as summary measures. In addition, the highest posterior density (HPD) intervals are considered as credible intervals for the model parameters.

**Fig. 2:**
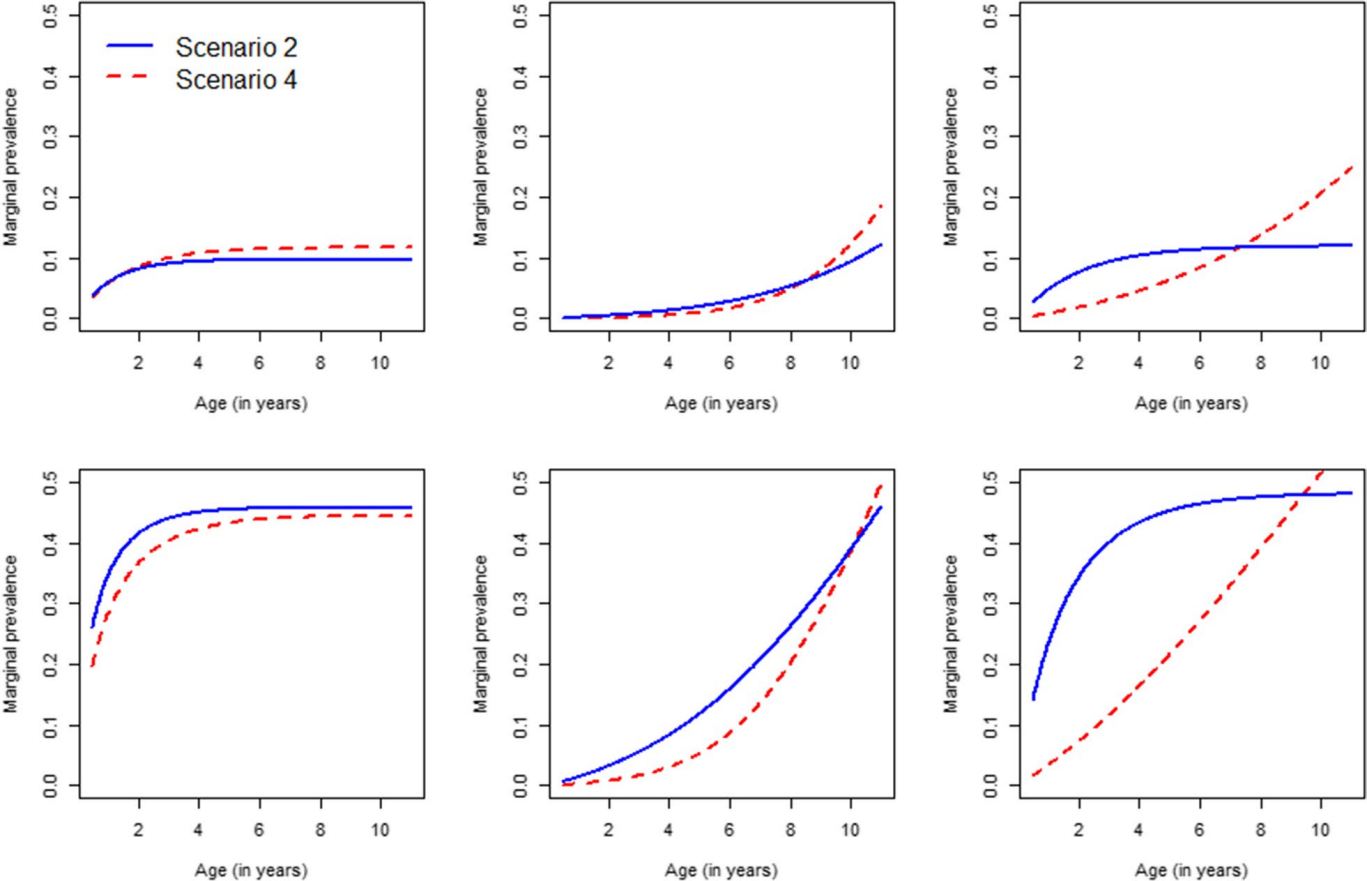
Estimated marginal prevalence for children assumed to be born in the baseline year (2001) by age, study site and symptomatic (top row) or asymptomatic (bottom row) at the previous visit, obtained by fitting a naive model ignoring ODS (while relying on routine and clinical data) (Scenario 2 model) and fitting the joint model which uses all data and accommodate ODS (Scenario 4 model). Left to right column: Walukuba, Kihihi and Nagongera

The posterior median parameter estimates and 95% HPD intervals for the converged samples from the models are displayed in Table 4, thereby having Gompertz baseline hazard functions $\lambda _0(a)$ and $\lambda _0^{*}(a)$ for the three study sites (see Additional file 1: Table B.1 in Appendix B for more details on the WAIC-values and deviance for the candidate models). A significant effect of shifted year of birth has been observed for Kihihi and Nagongera in both processes, and not for the low transmission intensity site Walukuba. The infection status at previous visit is included only for the outcome process resulting in an overall significant effect at all sites. In total, 35%, 43% and 62% of the observed visits were classified as clinical visits in Walukuba, Kihihi and Nagongera, respectively. Of those observed clinical visits, 87%, 48% and 54% are malaria-like clinical visits implying that no evidence of malaria infection was found in children coming to the clinic due to malaria-like symptoms. Figure 2 depicts the estimated marginal prevalence by age for children assumed to be born in the baseline year (2001) which were symptomatic (top row) or asymptomatic (bottom row) at the previous visit, and by study site (left to right: Walukuba, Kihihi and Nagongera). The curves are drawn for the Scenario 2 (solid blue line) and Scenario 4 (dashed red line) models. In general, the parasite prevalence increases with increasing age in areas with high (Nagongera) and medium (Kihihi) transmission intensity. In Walukuba, the prevalence first increases to a plateau from 6 months up to two years after the prevalence remains constant. From the graphs, it is clear that small differences exist between the two scenarios in terms of the estimated marginal prevalence.

**Fig. 3:**
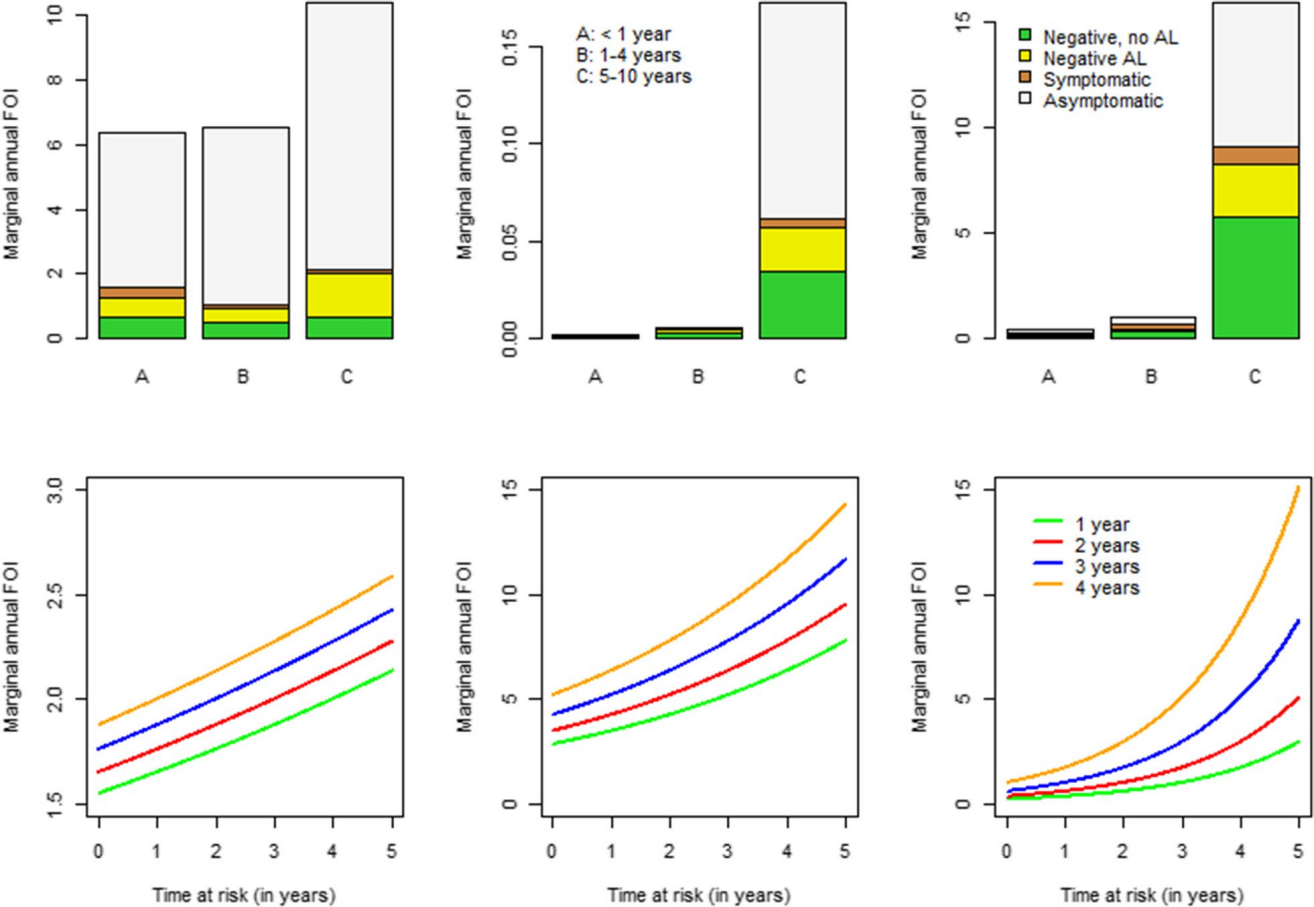
Estimated marginal FOI by time at risk and age when becoming at risk for the next malaria infection based on Scenario 4 and for children assumed to be born in the baseline year (2001). Top row: marginal FOI based on outcome process. Bottom row: marginal FOI based on time process. Left to right column: Walukuba, Kihihi and Nagongera

The estimated marginal annual FOI for the outcome (routine) process based on expression (2) is shown in Fig. 3. An annual parasite clearance rate ($\gamma$) of 1.643, 0.584 and 0.986 years$^{-1}$ for children aged less than 1 year, 1–4 years and 5–10 years is considered [21]. On top of that, the marginal annual FOI estimated from the time-to-event process is shown in the bottom row. The marginal annual FOI for the outcome process increases with increasing age in all study areas, and among children in age group 5–10 years and those that were previously asymptomatic (gray bars) and least in their symptomatic counterparts (brown bars). For the time process, an increase of the marginal annual FOI is also observed with increasing time at risk, and it is highest among children at a higher age in all study areas. More specifically, when children are older, the infection risk is high as compared to their younger counterparts given the specific time at risk. Unlike Walukuba and Kihihi districts, however, the marginal annual FOI in Nagongera is close to zero and constant with time at risk, at least for children aged 1 year when becoming at risk. For children at a higher age, the FOI tends to increase more rapidly with increasing time at risk and age.

## Discussion

In this paper, a new method has been proposed to account for ODS when estimating malaria transmission parameters such as, for example, the parasite prevalence and the FOI in case of longitudinal cohort data with routine (scheduled) and clinical (unscheduled) visits. A simulation study, inspired by parasitaemia data from a cohort of Ugandan children who were tested for malaria parasites (parasitaemia) during such visits, was conducted in which different parametric functions were considered to model the age-specific malaria prevalence and FOI while accounting for both observed and unobserved heterogeneity. The simulation results indicate that ignoring ODS tends to overestimate the marginal malaria infection risk and variance of the random effects. This is expected since children with a high disease activity are more likely to visit the clinic. Overestimation of such parameters may lead to incorrect inference and wrong conclusions. More specifically, from a public health point of view, ignoring ODS could potentially lead to overestimating the impact of efforts towards improved community-wide coverage of long-lasting insecticidal nets, indoor residual spraying of insecticides and additional malaria control interventions. Especially in settings where malaria elimination is achievable, reliable estimation of current malaria transmission is of crucial importance for the detection of malaria hotspots to be targeted. It has been demonstrated that the bias can be reduced by using a joint model in which both outcome (routine) and observation-time (clinical) components are present. In order to reduce the bias, the analysis performed here suggests that malaria events occurring within 35 days after a first malaria infection might be treated as being part of the same infection. This is supported by the results presented by Maiga et al. [19] and Ndiaye et al. [20]. Simulation results were based on a single MCMC chain with 10,000 iterations (burn-in 1500 iterations) which was found to be sufficient for convergence. Due to the additional complexity in the final data application, both in terms of fixed as well as random effects structure, more iterations were needed to achieve convergence.

The results regarding the data application show that both the malaria parasite prevalence and the FOI increase with increasing age in an areas of medium and high transmission intensities, Kihihi and Nagongera, respectively. In Walukuba which is an area of low transmission intensity, however, the prevalence peak at the age of about 2 years, after which remains constant. The FOI is highest in children aged 5–10 years and it becomes higher as children grow older or are at risk for a longer time. Unlike the areas of low (Walukuba) and medium (Kihihi) transmission intensities which were of high transmission intensity, the FOI in Nagongera is close to zero and constant with time at risk for children aged 1 year when becoming at risk. Further, both the prevalence and FOI are highest among the children with asymptomatic infections, and lower among the symptomatic ones or the previously treated children. These results are in line with those reported previously by Mugenyi et al. [5]. The high prevalence and FOI estimated among the older children particularly in area with high transmission is in agreement with the work by Doolan et al. [22]. The authors showed that children older than 5 years act as reservoirs for malaria parasites or asymptomatic infections and are rarely treated, hence leading to an increased infection risk.

One way to avoid bias in estimating the epidemiological parameters of interest is the use of routine data only. This approach has been demonstrated in the past [5]. However, the proposed methodology allows for a proper integration of clinical data in the data analysis, thereby enabling the study of potential different effects for symptomatic (detected at clinical visits) and asymptomatic (derived from routine data) infections. From the statistical analysis of the PRISM data performed here, the hypothesis of differential age-effects for symptomatic and asymptomatic infections is highly supported as models forcing the effects to be the same are clearly outperformed by their unrestricted and more flexible counterparts. Though the estimated parasite prevalence is in line with the observed data, more flexible parametric or semi-parametric baseline hazard functions could be considered in both processes which is an interesting avenue for further research. Furthermore, Mugenyi et al. [5] used a generalized linear mixed model to model the observed parasite prevalence after which the FOI is derived using equation (2). One of the shortcomings in this paper is the simplification of no parasite clearance when deriving the baseline hazard function for the time process. This could lead to an underestimation of the respective FOI. This is considered as an interesting avenue for future research.

The proposed joint model can be extended to have a (shared) parameter $\psi$ to allow for a non-trivial dependence between the outcome and observation time processes. More specifically, the dependence between the outcome and observation-time processes is governed by process-specific random effects $\varvec{b}_{i1}$ and $\varvec{b}_{i2}$, respectively (see, e.g, [14]), where $\varvec{b}_{i2} = \psi \varvec{b}_{i1}$. In that way, one can allow the process-specific random effects (both at the individual as well as household level) to act at different levels for both symptomatic as well as asymptomatic malaria episodes. However, applying this general approach to the PRISM data led to convergence issues, especially in those regions with low transmission intensity (Kihihi, Walukuba). A simplified model with only individual-level random effects, though different across processes (i.e., $\psi \ne 1$), however, did not outperform the model presented in the main text, including child- and household-specific random effects that are shared across the two processes (i.e., $\psi = 1$), except for Nagongera (high transmission intensity setting), while the significance of covariates was not altered (not shown here). The latter results from the presence of a high number of malaria episodes observed across various children in this study site.

To conclude, the combined model was applied to routine and clinical data on malaria infections from a cohort study in Uganda to estimate the parasite prevalence and FOI accounting for observed and unobserved heterogeneity, while dealing with ODS. Malaria parasite prevalence and FOI increase with age in the region of high malaria intensity with the highest FOI observed in age group 5–10 years. In all study sites, both the prevalence and FOI are highest among previously asymptomatic children and lowest among their symptomatic counterparts. Furthermore, inclusion of unscheduled clinical visits, which occur when individuals develop malaria-like symptoms, creates dependence between the study outcome and the observational time processes. Using a simulation study, the impact of ignoring this dependence and its potential to introduce bias in model parameter estimates was clearly demonstrated. This is confirmed by the fact that the combined model showed to have the smallest bias, especially when consecutive positive malaria parasitaemia presence results within a time period of 35 days were considered to be due to the same infection. Moreover, the proposed joint modelling approach can be used to model any longitudinal binary outcome data collected during scheduled routine observation times and linked to time to event data from the same subjects, obtained during unscheduled observation times.

## Supplementary Information

**Additional file 1.**Supplementary material to the manuscript including additional details concerning the simulation approach, fit andconvergence statistics for the PRISM data application and R code to fit the models.

## Data Availability

Access to the data analysed during the current study should be requested from the PRISM team.

## Abbreviations

PRISM: Program for Resistance, Immunology, Surveillance and Modeling of Malaria in Uganda
CI: Credible interval
MCMC: Markov Chain Monte Carlo
JAGS: Just Another Gibbs Sampler
WAIC: Watanabe-Akaike Information Criteria
AL: Artemether-Lumefantrine
FOI: Force of Infection
EIR: Entomological Inoculation Rate
RMSE: Root Mean Square Error
MSE: Mean Square Error
MLCI: Mean Length of Credible Intervals
ODS: Outcome Dependent Sampling
ODEs: Ordinary Differential Equations

## References

[1] World Health Organ (2016). Global malaria programme: World malaria report 2015.

[2] WHO. Malaria. World Health Organization; 2022. https://www.who.int/data/gho/data/themes/malaria.

[3] Smith DL, Drakeley CJ, Chiyaka C, Hay SI (2010). A quantitative analysis of transmission efficiency versus intensity for malaria. Nat Commun.

[4] Kamya MR, Arinaitwe E, Wanzira H, Katureebe A, Barusya C, Kigozi SP (2015). Malaria transmission, infection, and disease at three sites with varied transmission intensity in Uganda: implications for malaria control. Am J Trop Med Hyg.

[5] Mugenyi L, Abrams S, Hens N (2017). Estimating age-time-dependent malaria force of infection accounting for unobserved heterogeneity. Epidemiol Infect.

[6] Tan KS. Regression modeling of longitudinal outcomes with outcome-dependent observation times. Publicly Accessible Penn Dissertations, University of Pennsylvania. 2014;1467.

[7] Horvitz DG, Thompson DJ (1952). A generalization of sampling without replacement from a finite universe. J Am Stat Assoc..

[8] Holt D, Smith T (1980). Regression analysis of data from complex surveys. J R Stat Soc Ser A.

[9] Zhou H, Weaver MA, Qin J, Longnecker M, Wang M (2002). A semiparametric empirical likelihood method for data from an outcome-dependent sampling scheme with a continuous outcome. Biometrics.

[10] Park E, Kim Y (2004). Analysis of longitudinal data in case-control studies. Biometrika.

[11] Schildcrout JS, Heagerty PJ (2008). On outcome-dependent sampling designs for longitudinal binary response data with time-varying covariates. Biostatistics.

[12] Ryu D, Sinha D, Mallick B, Lipsitz SR, Lipshultz SE (2007). Longitudinal studies with outcome-dependent follow-up: models and Bayesian regression. J Am Stat Assoc.

[13] Rizopoulos D, Verbeke G, Molenberghs G (2008). Shared parameter models under random effects misspecification. Biometrika.

[14] Wulfsohn MS, Tsiatis AA (1997). A joint model for survival and longitudinal data measured with error. Biometrics.

[15] Pullenayegum EM, Lim LS (2016). Longitudinal data subject to irregular observation: a review of methods with a focus on visit processes, assumptions, and study design. Stat Methods Med Res.

[16] Mugenyi L, Nankabirwa JI, Arinaitwe E, Rek J, Hens N, Kamya M (2020). Estimating the optimal interval between rounds of indoor residual spraying of insecticide using malaria incidence data from cohort studies. PloS One.

[17] Watanabe S, Opper M (2010). Asymptotic equivalence of Bayes cross validation and widely applicable information criterion in singular learning theory. J Mach Learn Res.

[18] Plummer M. JAGS: A program for analysis of Bayesian graphical models using Gibbs sampling. In: Proceedings of the 3rd international workshop on distributed statistical computing. vol. 124. Vienna, Austria; 2003. p. 1–10.

[19] Maiga AW, Fofana B, Sagara I, Dembele D, Dara A, Traore OB (2012). No evidence of delayed parasite clearance after oral artesunate treatment of uncomplicated falciparum malaria in Mali. Am J Trop Med Hyg.

[20] Ndiaye JLA, Faye B, Gueye A, Tine R, Ndiaye D, Tchania C (2011). Repeated treatment of recurrent uncomplicated *Plasmodium falciparum* malaria in Senegal with fixed-dose artesunate plus amodiaquine versus fixed-dose artemether plus lumefantrine: a randomized, open-label trial. Malar J.

[21] Bekessy A, Molineaux L, Storey J (1976). Estimation of incidence and recovery rates of *Plasmodium falciparum* parasitaemia from longitudinal data. Bull World Health Organ.

[22] Doolan DL, Dobaño C, Baird JK (2009). Acquired immunity to malaria. Clin Microbiol Rev.

[23] Vehtari A, Gelman A, Gabry J (2017). Practical Bayesian model evaluation using leave-one-out cross-validation and WAIC. Stat Comput.

[24] Vehtari A, Gabry J, Magnusson M, Yao Y, Bürkner C Paul, Paananen T, et al. loo: Efficient leave-one-out cross-validation and WAIC for Bayesian models; 2020. R package version 2.3.1. https://mc-stan.org/loo/, https://discourse.mc-stan.org.

