# Supplementary material for: Modelling longitudinal binary outcomes with outcome dependent observation times: an application to a malaria cohort study
Source: Malar J. 2022 Dec 10;21:380. doi: 10.1186/s12936-022-04386-1 (PMC9741489; doi:10.1186/s12936-022-04386-1)
Supplement: Supplementary file 1 — Additional file 1.Supplementary material to the manuscript including additional details concerning the simulation approach, fit andconvergence statistics for the PRISM data application and R code to fit the models. [file 12936_2022_4386_MOESM1_ESM.pdf]

RESEARCH

Supplementary material to "Modelling longitudinal binary outcomes with outcome dependent observation times: An application to a malaria cohort study"

Adelino Martins[1,2], Sereina A. Herzog[3,4], Levicatus Mugenyi[6], Christel Faes[1], Niel Hens[1,4,5] and Steven Abrams[1,5*]

*Correspondence: steven.abrams@uhasselt.be
[1]Interuniversity Institute for Biostatistics and statistical Bioinformatics, Data Science Institute, UHasselt, Diepenbeek, Belgium
Full list of author information is available at the end of the article

Appendix

Appendix A: Simulation study

In this section we provide more details with regard to the simulation study for which a selection of the results are presented and discussed in the main text. In the simulation scenarios, the baseline malaria force of infection is considered to be constant, say λ_0. A general solution of the system of differential equations describing the disease dynamics in an SIS model is given by

$$I(a_{ij}|\boldsymbol{x}_i, \boldsymbol{b}_i) = I(0|\boldsymbol{x}_i, \boldsymbol{b}_i) \exp\left(-\int_0^a [\lambda_0(u) + \gamma]\, du\right)$$

Consequently, when modelling the parasite prevalence in the routine process with a generalized linear mixed model

$$g_l[I(a_{ij}|\boldsymbol{x}_i, \boldsymbol{b}_i)] = \beta_0 + \boldsymbol{b}_i,$$

where $g_l(\cdot)$ is a known link function, we assume that the parasite prevalence is independent of age and other covariates. Hence, expression 2 for the conditional force of infection simplifies to

$$\lambda(b_0) = \frac{\gamma g_l^{-1}(\beta_0 + \boldsymbol{b}_i)}{1 - g_l^{-1}(\beta_0 + \boldsymbol{b}_i)}.$$

The choice of the logit-link function implies a proportional hazards model since

$$\lambda(b_0) = \frac{\gamma \text{ expit}(\beta_0 + \boldsymbol{b}_i)}{1 - \text{expit}(\beta_0 + \boldsymbol{b}_i)} = \gamma \exp(\beta_0 + \boldsymbol{b}_i) = \gamma \exp(\beta_0) \exp(\boldsymbol{b}_i) = \lambda_0 \exp(\boldsymbol{b}_i),$$

with $\lambda_0 = \gamma \exp(\beta_0)$ and consequently $\beta_0 = \log(\lambda_0) - \log(\gamma)$. Hence, in our simulation approach, we consider the logit-link function in the generalized linear mixed model to implicitly estimate the underlying baseline force of infection based on the estimated parasite prevalence.

Table A.1 **Average number of malaria episodes, by varying percentage of assumed symptomatic infections (P). The labels C^+R^+ and R^+C^+ represent positive results at two near-by visits (C = clinical and R = routine) with the second observation deleted.**

	All data		Data for Scenario 4			
		Clinical	C^+R^+	R^+C^+		Clinical
**P**	N	%	%	%	N	%
20%	21687	7.8	0.2	0.03	21487	7.2
40%	22534	10.7	0.3	0.05	22334	10.6
60%	23376	14.8	0.6	0.06	23176	14.9
80%	24223	17.0	0.8	0.09	24077	17.5
100%	25077	19.4	0.9	0.11	24977	20.1

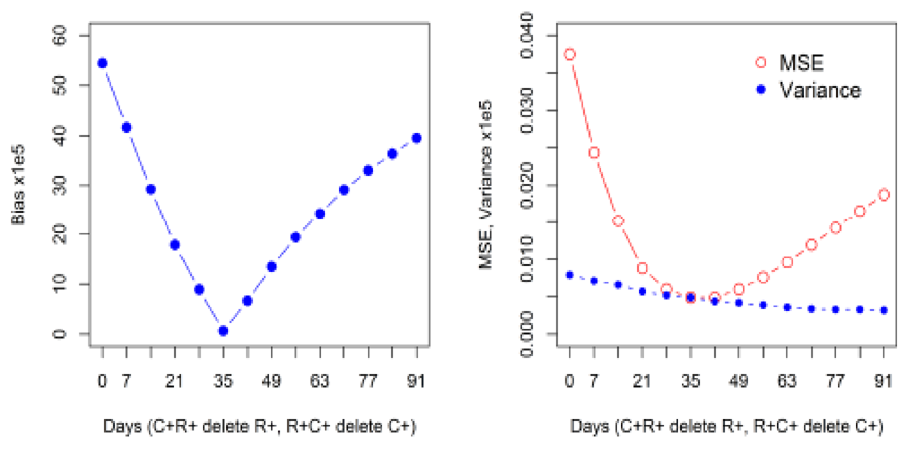

Figure A.1 Sensitivity analysis for bias, MSE and variance obtained using Scenario 4 by considering different number of days (1 week interval) between two consecutive visits with positive results. Bias and MSE are minimal if positive results observed within 35 days are considered to be of the same infection. C+ and R+ represent positive result/infection at clinical and routine visits, respectively.

Appendix B: Data application

B.1 Interval-censored infection times

Interval censoring occurs if the time at risk T_{AR} is only known to lie between two time points. In the PRISM study, the time to the second, third or the n-th infection is only known to lie between the point the child is tested positive and the point he/she first tested negative after recovering from the previous infection. Generally, if the real time at risk t_{AR} for the n-th infection of an individual of age a when becoming susceptible again at calendar time $t_{(n-1)}$, lies between t_L and t_U, then the probability density function for the time at risk is given by

$$\begin{aligned} f_{IC}(t_{AR}|a) &= P(t_L \leq T_{AR} \leq t_U|a) = F(t_U|a) - F(t_L|a) \\ &= S(t_L|a) - S(t_U|a), \end{aligned} \tag{B.1}$$

where $f_{IC}(t_{AR}|a)$ is the modified density function for interval-censored data (t_{AR}, a); $S(t_L|a)$ and $S(t_U|a)$ are the conditional survival functions evaluated in

t_L and t_U, respectively, i.e., for t_L,

$$S(t_L|a) = e^{-\int_a^{a+t_L} \lambda^*(u)du},$$

where $\lambda^*(u)$ is the infection hazard (for symptomatic infections). In case of **exponential** infection times, we have $\lambda^*(u) \equiv \lambda^*(u|\boldsymbol{x}) = \vartheta_1 e^{\boldsymbol{\zeta}'\boldsymbol{x}}$ and $S(t|a) = e^{-\vartheta_1 e^{\boldsymbol{\zeta}'\boldsymbol{x}} t}$, which implies

$$f_{IC}(t|a) = e^{-\vartheta_1 e^{\boldsymbol{\zeta}'\boldsymbol{x}} t_L} - e^{-\vartheta_1 e^{\boldsymbol{\zeta}'\boldsymbol{x}} t_U}.$$

Alternatively, for the **Weibull** and **Gompertz** distributions, it is straightforward to obtain similar expressions based on the expressions for the hazard functions in Table 1 in the main text. Finally, in case of the **fractional polynomial** model, we have $\lambda^*(u) \equiv \lambda^*(u|\boldsymbol{x}) = -\vartheta_2 u^{-2} e^{\vartheta_2 u^{-1}} e^{\boldsymbol{\zeta}'\boldsymbol{x}}$ and

$$S(t|a) = e^{-e^{\boldsymbol{\zeta}'\boldsymbol{x}}\left[e^{\vartheta_2(a+t)^{-1}} - e^{\vartheta_2 a^{-1}}\right]}.$$

Note that in case the first event recorded for an individual of age a is a clinical malaria infection, the time at risk lies in the interval $[t_L, t_U] = [t^o_{AR}, (a-\nu) + t^o_{AR}]$ where t^o_{AR} is the observed time at risk, a is the age of the individual at the entry of the study, and $0 \le \nu \le a$ is the age of the individual when becoming susceptible after the last infection prior to the inclusion into the study, thereby giving rise to a contribution $S(t^o_{AR}|a, \nu = 0) - S((a-\nu) + t_{AR}|a, \nu)$ to the likelihood function. Since ν is unknown, we need to marginalize over the probability density function of the random variable ν. However, this leads to complicated expressions for the likelihood function, hence, in this manuscript, we take $S(t^o_{AR}|a) - S(a + t^o_{AR}|0)$ as likelihood contribution, implying that $[t_L, t_U] = [t^o_{AR}, a + t^o_{AR}]$, and we consider the aforementioned marginalization strategy as further research which is beyond the scope of this paper. Hereunder, we describe 4 possible situations for the treatment of interval censoring in the PRISM study. First, let $t_{(n)}$ be the calendar time at which one tests positive for the n-th infection ($n > 1$), $t_{(n-1)}$ the point at which one first tests negative from the $(n-1)$-th infection, and $t^*_{(n-1)}$ be the calendar time one was last observed positive for the $(n-1)$-th infection.

Situation 1: If $t_{(n-1)}$ and $t_{(n)}$ are exactly the points when one becomes susceptible and infected, respectively, then time at risk, $t_{AR} = t_{(n)} - t_{(n-1)}$. In this case there is no interval censoring and the contribution to the likelihood is simply $f(t_{AR}|a)$, where a is the age of the individual at time $t_{(n-1)}$.

Situation 2: If $t_{(n-1)}$ is exactly the point when one becomes susceptible, then the time at risk, $t_{AR} \in [0, t_{(n)} - t_{(n-1)}]$, meaning that $t_L = 0$ and $t_U = t_{(n)} - t_{(n-1)}$. Consequently, a represents the age of the individual at time $t_{(n-1)}$ in likelihood contribution (B.1).

Situation 3: If $t_{(n)}$ is exactly the point when one becomes infected, then the time at risk, $t_{AR} \in [t_{(n)} - t_{(n-1)}, t_{(n)} - t^*_{(n-1)}]$, meaning that $t_L = t_{(n)} - t_{(n-1)}$, $t_U = t_{(n)} - t^*_{(n-1)}$ and a represents the age of the individual at calendar time $t^*_{(n-1)}$.

Situation 4: If $t^*_{(n-1)}$ is exactly the point when one becomes susceptible, then the time at risk, $t_{AR} \in [0, t_{(n)} - t^*_{(n-1)}]$, meaning that $t_L = 0$, $t_U = t_{(n)} - t^*_{(n-1)}$ and a

represents the age of the individual at calendar time $t^*_{(n-1)}$.
The statistical analysis presented in this paper is based on Situation 2, though the other situations are also plausible and worth considering, albeit that these scenarios are all approximations of the truth. The impact of assuming Scenarios 3–4 on inference was found to be minor and the conclusions did not change.

B.2 Fit statistics

Table B.1 Fit statistics for models fitted to PRISM data based on Scenario 4 by study site. Better fits for each site based on WAIC are indicated in bold.

Site	Fit statistic	Exponential	Weibull	Gompertz	Fractional polynomial	Log-logistic
Walukuba:	WAIC	1780.84	1194.68	**1192.14**	1364.45	1242.19
	Deviance	1656.312	1057.23	1052.36	1210.11	1090.87
Kihihi:	WAIC	6659.53	4082.86	**4057.80**	5371.73	4185.07
	Deviance	6368.50	3834.92	3805.34	5060.21	3943.02
Nagongera:	WAIC	8568.24	4717.80	**4638.65**	6380.89	4652.80
	Deviance	7919.55	4424.30	4369.31	6009.22	4385.24

Appendix C: Model convergence diagnostic

C.1 Walukuba

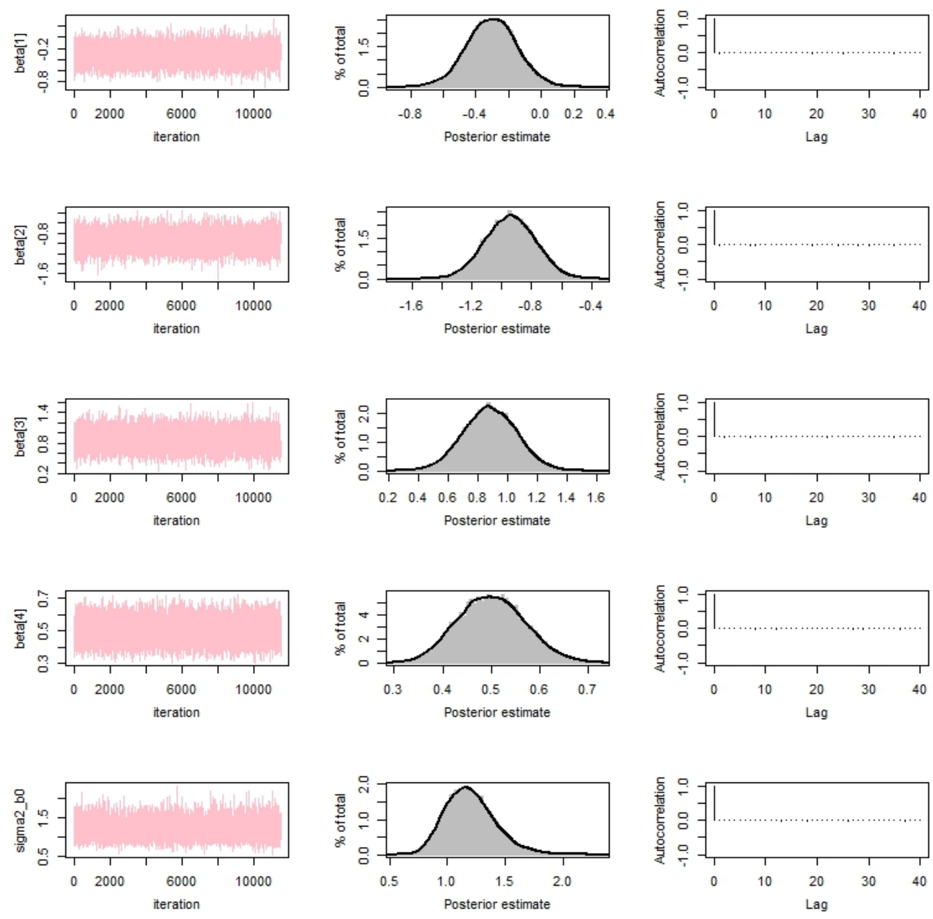

Figure C.1 Model convergence diagnostics: Trace plots (left panel), posterior distribution (middle panel) and auto-correlation plots (right panel). Convergence achieved after 250,000 thinned by a factor of 15 so that the remaining 14,000 samples was used to estimate the posterior distributions of the model parameters-Wulukuba site.

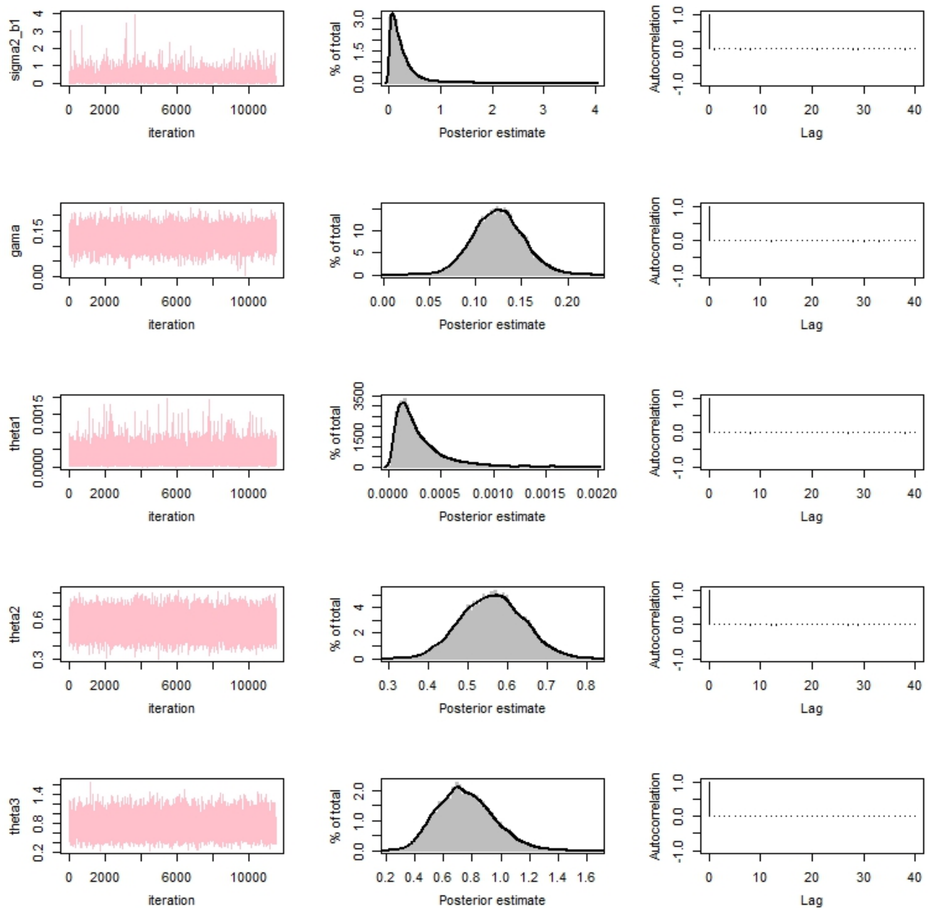

Figure C.2 Model convergence diagnostics: Trace plots (left panel), posterior distribution (middle panel) and auto-correlation plots (right panel). Convergence achieved after 250,000 thinned by a factor of 15 so that the remaining 14,000 samples was used to estimate the posterior distributions of the model parameters-Wulukuba site.

C.2 Kihihi

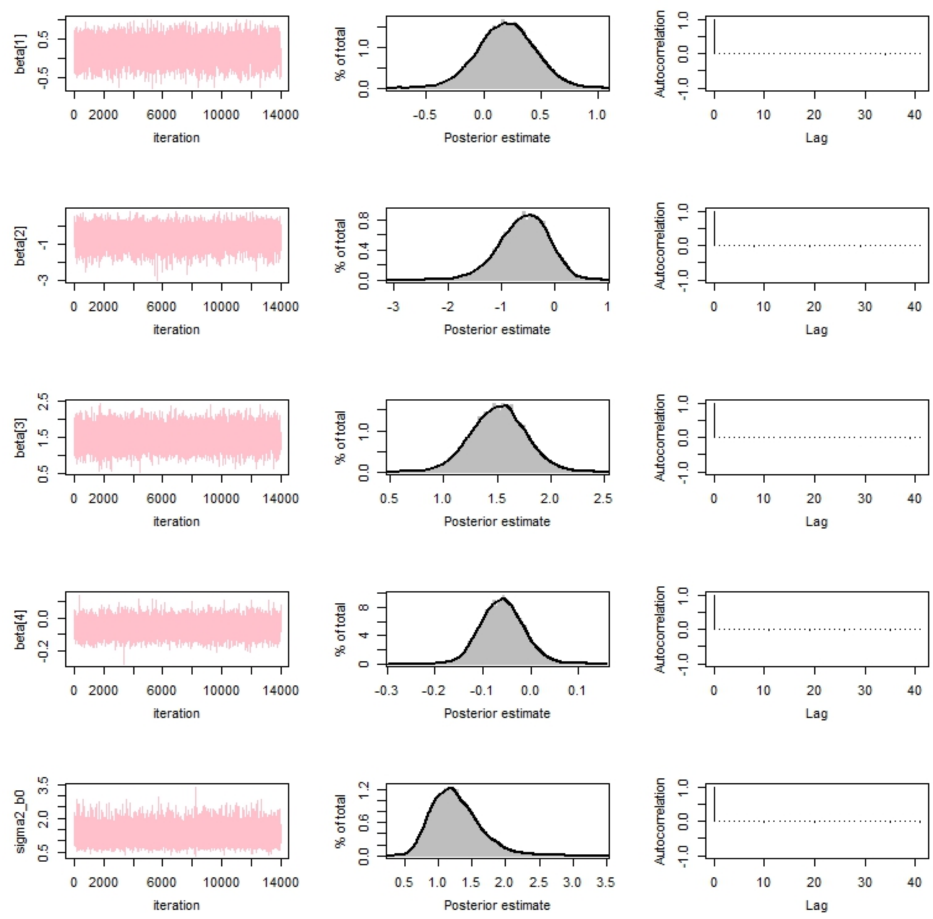

Figure C.3 Model convergence diagnostics: Trace plots (left panel), posterior distribution (middle panel) and auto-correlation plots (right panel). Convergence achieved after 250,000 thinned by a factor of 15 so that the remaining 14,000 samples was used to estimate the posterior distributions of the model parameters-Kihihi site.

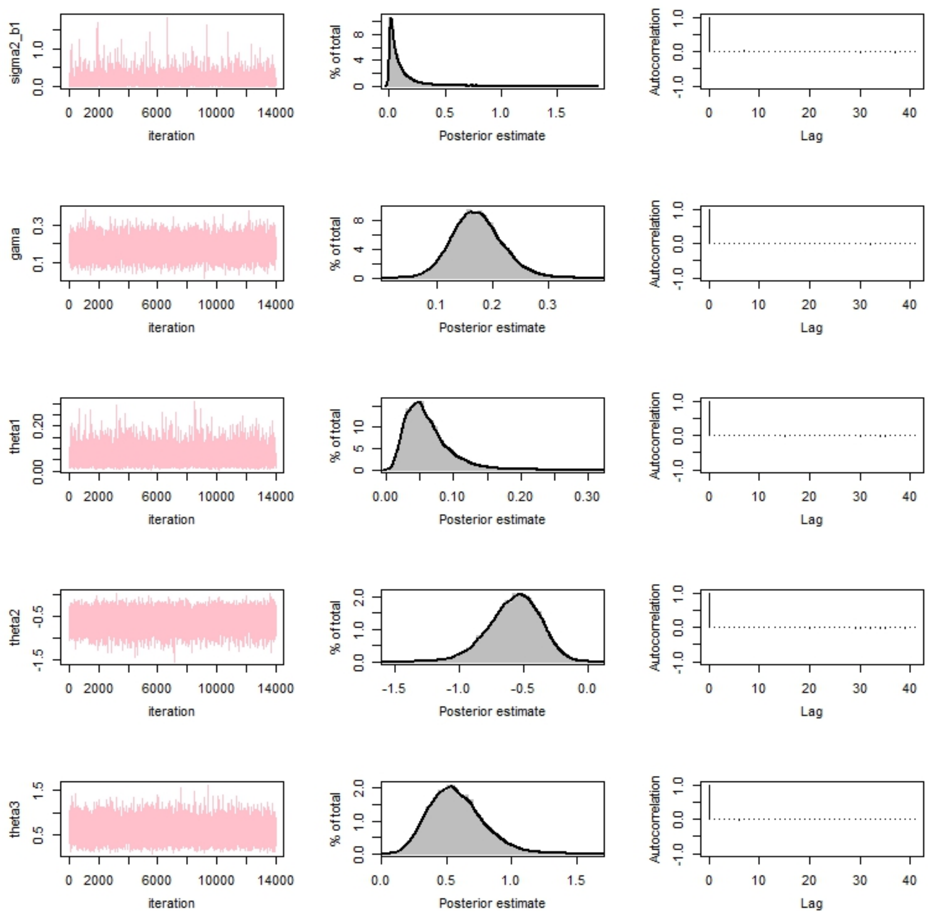

Figure C.4 Model convergence diagnostics: Trace plots (left panel), posterior distribution (middle panel) and auto-correlation plots (right panel). Convergence achieved after 250,000 thinned by a factor of 15 so that the remaining 14,000 samples was used to estimate the posterior distributions of the model parameters-Kihihi site.

C.3 Nagongera

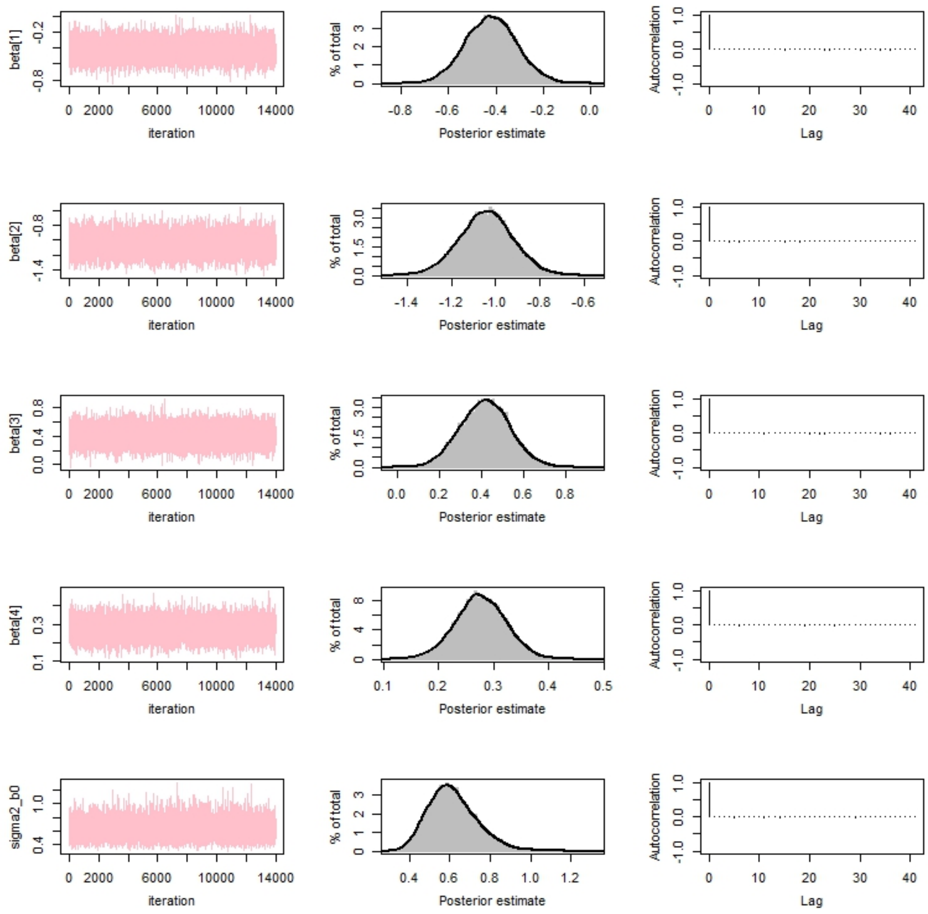

Figure C.5 Model convergence diagnostics: Trace plots (left panel), posterior distribution (middle panel) and auto-correlation plots (right panel). Convergence achieved after 250,000 thinned by a factor of 15 so that the remaining 14,000 samples was used to estimate the posterior distributions of the model parameters-Nagongera site.

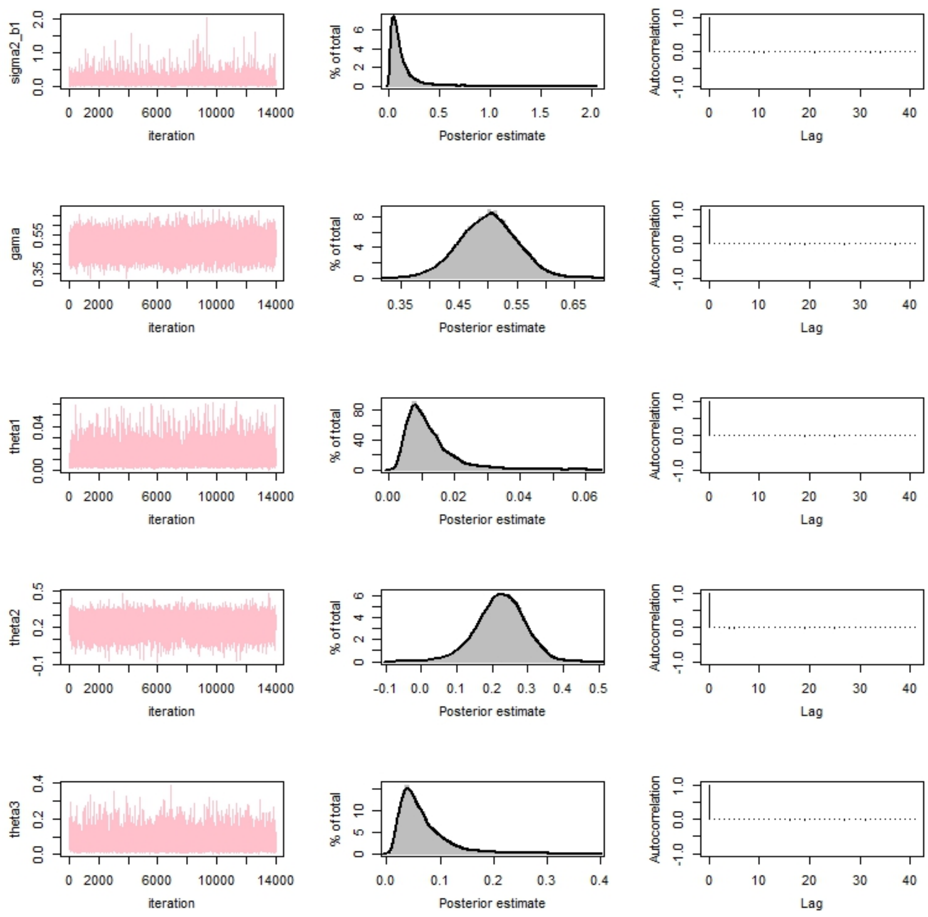

Figure C.6 Model convergence diagnostics: Trace plots (left panel), posterior distribution (middle panel) and auto-correlation plots (right panel). Convergence achieved after 250,000 thinned by a factor of 15 so that the remaining 14,000 samples was used to estimate the posterior distributions of the model parameters-Nagongera site.

Appendix D: R code to fit the models

```
=================================================================
writeLines("
model{
C <- 10000
for(i in 1:N){
zeros[i] ~ dpois(l[i]);l[i] <- -logLike[i]+C
# Linear predictor
lp1[i] <- beta[1]*NegativeAL[i]+beta[2]*Symptomatic[i]+beta[3]*
          Asymptomatic[i]+beta[4]*ysh[i]
lp2[i] <- gama*ysh[i]
ha[i] <- log((theta1[1]/theta1[2])*(exp(theta1[2]*age[i])-1))
R[i] <- b0[hhid[i]]+b[hhid[id[i]]]
cloglog(p[i]) <- max(-20,min(20,ha[i]+lp1[i]+R[i]))
fLT1[i]<-max(1e-300,exp(-(theta2[1]/theta2[2])*exp(lp2[i]+
         R[i])*(exp(theta2[2]*(age[i]+trisk[i]))-
         exp(theta2[2]*age[i])))-exp(-(theta2[1]/theta2[2])
         *exp(lp2[i]+R[i])*(exp(theta2[2]*(age[i]+trisk[i]))
         -1)))
fIC1[i]<-max(1e-300,1-exp(-(theta2[1]/theta2[2])*exp(lp2[i]+
         R[i])*(exp(theta2[2]*(age[i]+trisk[i]))-
         exp(theta2[2]*age[i]))))
St1[i]<-max(1e-300,1-exp(-(theta2[1]/theta2[2])*exp(lp2[i]+
        R[i])*(exp(theta2[2]*(age[i]+TTime[i]))-
        exp(theta2[2]*age[i]))))
I1[i] <- ifelse(tau[i] == 1, 1, 0)
fLT[i] <- I1[i]*fLT1[i]+(1-I1[i])
I2[i] <- ifelse(tau3[i] == 0, 1, 0)
fIC[i] <- I2[i]*fIC1[i]+(1-I2[i])
St[i] <- I2[i]*St1[i]+(1-I2[i])
# Log likelihood function
logLike[i]<-delta[i]*(tau[i]*log(fLT[i])+(1-tau[i])*((1-tau2[i])*
            log(fIC[i])+tau2[i]*log(fIC[i]/St[i])))+(1-delta[i])*
            (paras[i]*log(p[i])+(1-paras[i])*log(1-p[i]))
     }
# Household random effect
for(j in 1:n){b0[j] ~ dnorm(mus0,tau.s0)}
# Child random effect
for(k in 1:m){b[k] ~ dnorm(mus1,tau.s1)}
tau.s0 ~ dgamma(0.01,0.01);tau.s1 ~ dgamma(0.01,0.01)
mus0 <- -inverse(tau.s0)/2;mus1 <- -inverse(tau.s1)/2
# variance of the household random effect
sigma2b0 <- inverse(tau.s0)
# variance of the child random effect
sigma2b1 <- inverse(tau.s1)
# Prior distribution for fixed effect
```

```
for(l in 1:4){beta[l] ~ dnorm(0,0.001)}
gama ~ dnorm(0,0.001)
# Prior distribution for baseline hazard parameters
theta1[1] ~ dgamma(0.01,0.01);theta1[2] ~ dnorm(0,0.001)
theta2[1] ~ dgamma(0.01,0.01);theta2[2] ~ dnorm(0,0.001)
   }
           ", con="modelODS.txt")
modfile.ODS <- 'modelODS.txt'
===============================================================
```

Author details
[1]Interuniversity Institute for Biostatistics and statistical Bioinformatics, Data Science Institute, UHasselt, Diepenbeek, Belgium. [2]Department of Mathematics and Informatics, Eduardo Mondlane University, Maputo, Mozambique. [3]Institute for Medical Informatics, Statistics and Documentation (IMI), Medical University of Graz, Graz, Austria. [4]Centre for Health Economics Research and Modelling Infectious Diseases, Vaccine and Infectious Disease Institute, University of Antwerp, Antwerp, Belgium. [5]Family Medicine and Population Health, University of Antwerp, Antwerp, Belgium. [6]Infectious Diseases Research Collaboration, Plot 2C Nakasero Hill road, Kampala, Uganda.

References
